# Addressing challenges of training a new generation of clinician-innovators through an interdisciplinary medical technology design program: Bench-to-Bedside

**DOI:** 10.1186/s40169-015-0056-3

**Published:** 2015-04-19

**Authors:** Patrick D Loftus, Craig T Elder, Troy D’Ambrosio, John T Langell

**Affiliations:** Center for Medical Innovation, University of Utah, 10 North 1900 East, Spencer S. Eccles Health Sciences Library Room 15, Salt Lake City, Utah 84132 USA; Lassonde Entrepreneur Institute, University of Utah, 105 Fort Douglas, Bldg. #604, Salt Lake City, Utah 84113 USA

**Keywords:** Bench-to-Bedside, Clinical innovation, Medical competition, Research paradigm, Industry, Market, Publishing papers, Medical education

## Abstract

Graduate medical education has traditionally focused on training future physicians to be outstanding clinicians with basic and clinical science research skills. This focus has resulted in substantial knowledge gains, but a modest return on investment based on direct improvements in clinical care. In today’s shifting healthcare landscape, a number of important challenges must be overcome to not only improve the delivery of healthcare, but to prepare future physicians to think outside the box, focus on and create healthcare innovations, and navigate the complex legal, business and regulatory hurdles of bringing innovation to the bedside. We created an interdisciplinary and experiential medical technology design competition to address these challenges and train medical students interested in moving new and innovative clinical solutions to the forefront of medicine. Medical students were partnered with business, law, design and engineering students to form interdisciplinary teams focused on developing solutions to unmet clinical needs. Over the course of six months teams were provided access to clinical and industry mentors, $500 prototyping funds, development facilities, and non-mandatory didactic lectures in ideation, design, intellectual property, FDA regulatory requirements, prototyping, market analysis, business plan development and capital acquisition. After four years of implementation, the program has supported 396 participants, seen the development of 91 novel medical devices, and launched the formation of 24 new companies. From our perspective, medical education programs that develop innovation training programs and shift incentives from purely traditional basic and clinical science research to also include high-risk innovation will see increased student engagement in improving healthcare delivery and an increase in the quality and quantity of innovative solutions to medical problems being brought to market.

## Background

Traditional clinical and basic science research forms the foundation of medical care and medical education. Recognized shortfalls in how graduate medical education has been delivered have resulted in many medical schools adopting a more integrated curricular approach to teaching [1,2]. However, without the development of clinician-driven innovation, many of the advances made through these teaching approaches remain unrealized – often never leaving the laboratory bench [3]. As the demand for better and more cost effective health care quality and delivery continues to increase – expensive, limited, and time consuming traditional research programs are put under greater scrutiny to produce outputs not necessarily consistent with their primary goals [3,4]. Furthermore, the current pathway for training clinician-researchers and clinician-innovators is riddled with needs and challenges [3,5-7]. Attempts at addressing these issues have included combined degrees (MD/MBA), which between 2011 and 2012 alone, the number of MD/BMA programs increased by 25%, as well as interdisciplinary programs such as Stanford Biodesign [8,9]. These programs are extremely valuable but often require more time away from medical education than most medical students will accept, and raise the concern for drawing down the number of clinicians who participate in clinical care in an environment with an ever increasing need for more healthcare providers [8].

In the viewpoint, “Health Services Innovation: The Time is Now”, Zuckerman *et al.* explicitly identified a number of challenges to training a new generation of clinician-innovators [3]: First, hospital and medical school leadership need to value and promote understanding of systems of care and innovation; Second, training in technical methods for innovation…will need to come from nontraditional places – business, engineering, and design schools. Strategies from industry, such as innovation challenges and “crowd sourcing”, will further enhance development of new ideas; Third, the traditional incentives in academia to reward publications and funding for traditional research will need to be adjusted to include incentives for clinician-innovators and to have greater tolerance of risk because most innovations will fail.

Herein, we present our solution to bridge the gap in clinician-innovation training. We provide data from a four–year experience with a student-driven, experiential medical device design competition, entitled Bench-to-Bedside, that engages stakeholders at all levels to support a paradigm shift in the driving incentives of medical education from developing traditional clinician-researchers to developing clinician-innovators. We believe this paradigm shift will play a valuable role in translational research, which deals with the awareness, acceptance and adoption of new technology, with adoption often being the most difficult component [10]. Studies have shown clinical innovations built on evidence-based medicine are more likely to be adopted [11], and the speed of adoption is dependent on senior management and clinical leadership [12]. By placing clinical leadership, starting at the level of the medical student, at the forefront of translational medicine through an interdisciplinary innovation program we anticipate more clinical innovations will be brought to market than programs built solely on basic science research and/or healthcare management. The program we have created addresses the main innovation challenges identified by Zuckerman *et al.* [3], provides a model for translational research implementation, and gives us an experiential perspective we hope to share with others.

The detailed outline of the organization, structure and curriculum of the competition can be found in the *OPEN Conference Report* [13]. In brief, medical students were partnered with graduate students from business, engineering, design and law, provided $500 in prototype funding, and given 6 months to develop a solution to an unmet clinical need, thus bridging the gap from discovery to clinical delivery anticipated in translational research [14]. The primary outcomes evaluated included yearly participants, medical student participants, and teams. Secondary outcomes included devices developed, provisional patents filed, utility patents filed, and LLCs formed. The outcomes were evaluated from year-to-year totals. Success was determined by an increasing number of secondary outcomes from year to year. The initial recruitment goal for the first year was five teams. Each of the clinician-innovation training challenges were addressed as follows:

### First challenge: establishing innovation value by medical leadership

In June 2010, the Dean of the School of Medicine at the University of Utah announced the creation of a new Center for Medical Innovation to house the B2B competition, along with other newly developed University of Utah medical innovation programs. The center formalized partnerships between the College of Engineering, the colleges in the Health Sciences Center, the School of Business, the College of Law, and the ongoing Technology Venture Development Program. The Center for Medical Innovation managed outside sponsorship of the B2B competition, financial support of student projects, and established guidelines and resources to help prevent students from entering into inappropriate and conflicting agreements with outside pharmaceutical and medical device companies.

### Second challenge: developing nontraditional training – business, engineering and design

Bench-to-Bedside was designed to leverage interdisciplinary education through the formation of entrepreneurial teams composed of medical students, engineering students, business students, and law students – who form start-up companies (Table 1). The teams are given the task of identifying an unmet clinical need, as well as access to many resources including: university physicians mentors, technology development funds, the Lassonde Entrepreneurship Institute, and the Center for Medical Innovation intellectual property and new venture law fellows. Workshops are held covering clinical needs identification, business plan development, prior art searching, FDA regulatory compliance, engineering and design prototyping, patent development, and term sheet negotiations (Table 1) [13].

**Table 1:** **Nontraditional training: list of disciplines medical students are exposed to via interdisciplinary teams, as well as yearly workshops, in the Bench-to-Bedside Competition**

**Medical student exposure to non-traditional training**	
**By discipline (Team)**	**By topic (Workshop)**
Health Sciences:	Clinical Need Identification
• Biomedical Informatics	Business Plan Development
• Nursing	Prior Art Searching
• Pharmacy	FDA Regulations
• Exercise & Sports Science	Prototyping
Business:	Term Sheet Negotiation
• Business Administration	Patent Development
• Entrepreneurship	Venture Capital
Engineering:	
• Biomedical Engineering	
• Mechanical Engineering	
• Electrical Engineering	
• Entertainment Arts & Engineering	
• Chemical Engineering	
• Materials Engineering	
Basic Science:	
• Chemistry	
• Biology	
• Life Sciences	
• Mathematics	
• Genetics	
Other:	
• Law	
• Computer Science	
• Art Education	
• Architecture	
• Film & Media Arts	
• English	

### Third challenge, part A: applying strategies from industry

To provide further support and training, the Center for Medical Innovation established an Industrial Board composed of community leaders in the medical device industry. Each team is paired with one of these leaders as a mentor, who provides direction and advice throughout the competition. Furthermore, at the end of the competition, many of the mentors continue to educate and support the teams through the commercialization process.

### Third challenge, part B: providing crowdsourcing and milestone funding

While the primary purpose of the competition is to educate future clinician-innovators, a milestone-funding program was established to promote continued development of ideas in the years following completion of the competition. B2B partnered with the University of Utah Entrepreneur Club to provide ongoing financial support to teams continuing beyond competition night. Student teams make milestone pitches to the Entrepreneur Club, which validates the ideas and determines financial support needed to reach their next milestone. As ideas are appropriately validated, B2B provides the funds necessary for teams to advance the commercialization of their technologies.

### Fourth challenge: adjustment of incentives

In order to better prepare medical students for a meaningful career in medicine, the University of Utah School of Medicine requires each medical student to complete a scholarly activity – constituting a hypothesis based research project. The basis of this requirement has roots based on the incentives of academia to reward publications and funding for traditional research. After demonstrating during the first three years of the competition that most, if not all, of the clinical innovations were hypothesis based, the Bench-to-Bedside student leadership proposed to the University of Utah School of Medicine curriculum committee a change to the scholarly activity requirement to include clinical innovation. After three attempts, and from support of the dean of the medical school, the curriculum committee supported an adjustment in its policy to accept medical device innovation as a fulfillment of the scholarly activity requirement, along with both clinical and basic science research. The University of Utah School of Medicine determined that completion of the Bench-to-Bedside program was adequate to replace the traditional research project based on four principles: First, the program allowed students to find and follow a passion that helped distinguish them throughout medical school and beyond. Second, students were able to develop expertise in not only one or more areas of medicine, but also in engineering, business, and law. Third, students through the translational process were able to perform inquiry-based research via needs-identification and design iteration. Fourth, through milestone funding and the awards night of the competition, students were able to effectively communicate the rationale for and results of their innovative activity. Through each of these principles students were completing the same tasks of traditional research, but instead of a publication at the end of the year, they had a new innovation.

## Outcomes and discussion

After four years of implementation, the competition supported 396 participants (144 of which were medical students) and 87 teams, which developed 91 new medical devices and launched 24 new companies (Table 2). A total of 55 provisional patents were filed, 15 of which have made it to utility patents. Secondary outcomes including devices developed, provisional patents filed, utility patents filed, and LLCs formed increased 3.1, 1.3, 5, and 12 fold, respectively, from the first year to the fourth year of competition, demonstrating that as solutions to the initial four challenges identified by Zuckerman *et al.* were applied, improved secondary outcomes were seen.

**Table 2:** **Participant, team, device, patent, and company totals for each year of the competition**

**Competition results**					
	**2011**	**2012**	**2013**	**2014**	**Totals**
Yearly Participants	76 (19%)	57 (14%)	74 (19%)	189 (48%)	396 (100%)
Medical Student Participants	32 (22%)	23 (16%)	36 (25%)	53 (37%)	144 (100%)
Teams	13 (15%)	14 (16%)	18 (21%)	42 (48%)	87 (100%)
Devices Developed	14 (15%)	14 (15%)	20 (22%)	43 (48%)	91 (100%)
Provisional Patents Filed	12 (22%)	13 (24%)	14 (25%)	16 (29%)	55 (100%)
Utility Patents Filed	1 (7%)	4 (27%)	5 (33%)	5 (33%)	15 (100%)
LLCs Formed	1 (4%)	4 (17%)	7 (29%)	12 (50%)	24 (100%)

Percentages of the total are listed. LLCs = Limited Liability Companies.

It was not until the adjustment of incentives in late 2013 allowing B2B projects to fulfill the scholarly activity requirement, as well as the establishment of milestone funding to continuing teams and pairing of students with industry mentors, that a marked increase in student participation was observed. The number of medical students participating in 2014 increased approximately 1.5 fold to a total of 53. Anecdotally, the increase in participating medical students led to a 2.5 fold increase in interdisciplinary student participation – the medical students often being the crux of identifying true clinical needs and maintaining teams.

With the establishment of milestone funding to continuing teams and industry mentors, not only did new participants enter the competition, but students who competed in previous years returned with advanced experience – increasing the number of limited liability companies (LLCs) and devices (Table 2). For a complete list of clinical problems and solutions developed by student teams over the program’s history, as well as success stories for projects being adopted into market, please refer to our published competition reports [15,16], as well as the Bench-to-Bedside website for the most recent devices (http://healthsciences.utah.edu/center-for-medical-innovation/students/bench-to-bedside.php).

The B2B competition would have long remained a small student event if the medical school leadership had not seen the value of student innovation education. When the first challenge of establishing value by medical leadership was met and a center for medical innovation was established, it became easier to develop a nontraditional training competition for medical students – which included business, engineering, and design exposure.

Through the Center for Medical Innovation, industry-university relationships were nurtured and applied to students. Pairing of students with business mentors, providing milestone funding, and adjusting incentives to support high-risk innovation showed exponential growth of competition participants, medical devices produced, limited liability companies, and patents.

While we have experienced extremely optimistic outcomes in training a new generation of clinician-innovators, we recognize there is yet a long way to travel in the paradigm shift from the incentives of academia to reward publications and funding for traditional research to reward often high risk innovations. Some of these initial steps include collecting data concerning perceptions of innovation importance from residency programs and student interviews. Furthermore, the developed devices from the B2B Competition will require long-term follow up to evaluate the true worth of an extracurricular interdisciplinary program. Students developing their education through hands-on nontraditional learning experiences will need to be assessed compared to traditional classroom learning for knowledge output and capability to succeed in the clinical environment. Furthermore, evaluation of reasons for student participation will be continuously required.

Although these next steps certainly exist, their unknown and ongoing answers do not diminish the need to truly develop a new generation of clinician innovators. Furthermore, they do not distract from the program’s highly positive innovation outcomes. While many business competitions exist for students, many of which can be found on istart.org, we are unaware of any competition that is focused primarily on medical innovation and the training of clinician innovators through an interdisciplinary approach involving business, law, engineering, and design.

## Conclusion

Our four-year outcomes data has validated the program’s approach as a model for training future clinician-innovators and as a benchmark translational research implementation program, resulting in 24 new companies and the creation of 91 new medical devices. Furthermore, our outcomes validated our solutions to the challenges proposed by Zuckerman et al. We believe there are several critical lessons learned from our experience that can be applied to other future programs, all of which will help bridge the gap of training clinician innovators and bringing solutions from the bench to the bedside.

First Lesson: a subset of medical students is inherently interested in non-traditional training (business, engineering, design). When medical school leadership value medical innovation by creating opportunities, such as innovation centers and competitions, as well as establishing resources for product development, their initial setup risk will be compensated by increased student interest and, linearly, increased student outputs. Furthermore, these centers and competitions will serve as the basis for business, engineering, and design training of future clinician-innovators.

Second Lesson: opening the doorway for students to interact with industry professionals in a safe manner that does not compromise or conflict with their education provides hands-on innovation training that cannot be taught in the classroom, creates an invaluable network of university-industry connections, and increases the likelihood of medical solutions succeeding – as evidenced by B2B’s increase in both limited liability companies and patents during the fourth year of the competition. The benefits of such university-industry connections are not new [17], however their extension to the current training of future clinicians shows extreme promise towards student retention, project advancement, and the creation of long-term economic benefit.

Third Lesson: the most important factor for increasing participation in a non-mandatory innovation-training program was to move the incentive focus from traditional research, including publishing papers, to potentially high-risk experimental innovation. In the most recent year of the competition, this adjustment to the University of Utah’s scholarly activity requirement has close to doubled the number of medical student participants (Table 2). In a “publish or perish” environment, hospital and medical curriculum leadership, must be willing to redefine what makes a great student, resident, or clinician when innovations fail [18]. By having a scholarly activity requirement that can be met, by not only basic and clinical science research and publications, but also development of a new medical device – even if the device fails in the market – a student can have confidence that their long-term residency and employment risks, which often appear dependent on publications instead of innovations, can be mitigated.

We agree with Zuckerman *et al.* wherein they stated [3]*, “*A new generation of trained clinician-innovators is needed to identify opportunities for innovation when others see problems and frustration. These clinicians will need support to initiate the small but important changes that may ultimately lead to larger transformations in health care”. The lessons we have learned, herein, provide many of the starting points for initiation of these small but important changes. Their application will open the doorway for an innovation-led paradigm shift of the up and coming generation towards better healthcare delivery and translational research.

## Abbreviation

B2B: Bench-to-Bedside
